# *In silico* identification of human microRNAs pointing centrin genes in *Leishmania donovani*: Considering the RNAi-mediated gene control

**DOI:** 10.3389/fgene.2023.1329339

**Published:** 2024-02-08

**Authors:** Manei M. Aljedaie, Pravej Alam

**Affiliations:** Department of Biology, College of Sciences and Humanities, Prince Sattam Bin Abdulaziz University, Al-Kharj, Saudi Arabia

**Keywords:** *Leishmania donovani*, centrin genes, calcium-binding proteins, *in silico*, microRNAs, miRDB, STRING

## Abstract

Leishmaniasis, a parasitic disease caused by different species of the protozoa parasite *Leishmania*, is a neglected tropical human disease that is endemic in about a hundred countries worldwide. According to the World Health Organization (WHO), the annual incidence of cutaneous leishmaniasis (CL) is estimated to be 0.7–1.2 million cases globally, whereas the annual incidence of visceral leishmaniasis is estimated to be 0.2–0.4 million cases. In many eukaryotic organisms, including human beings and protozoan parasites, centrin genes encode proteins that play essential roles within the centrosome or basal body. Human microRNAs (miRNAs) have been linked to several infectious and non-infectious diseases associated with pathogen–host interactions, and they play the emphatic roles as gene expression regulators. In this study, we used the MirTarget bioinformatics tool, which is a machine learning-based approach implemented in miRDB, to predict the target of human miRNAs in *Leishmania donovani* centrin genes. For cross-validation, we utilized additional prediction algorithms, namely, RNA22 and RNAhybrid, targeting all five centrin isotypes. The centrin-3 (LDBPK_342160) and putative centrin-5 (NC_018236.1) genes in *L. donovani* were targeted by eight and twelve human miRNAs, respectively, among 2,635 known miRNAs (miRBase). hsa-miR-5193 consistently targeted both genes. Using TargetScan, TarBase, miRecords, and miRTarBase, we identified miRNA targets and off-targets in human homologs of centrin, inflammation, and immune-responsive genes. Significant targets were screened based on GO terminologies and KEGG pathway-enrichment analysis (Log10 *p*-value >0.0001). *In silico* tools that predict the biological roles of human miRNAs as primary gene regulators in pathogen–host interactions help unravel the regulatory patterns of these miRNAs, particularly in the early stages of inflammatory responses. It is also noted that these miRNAs played an important role in the late phase of adaptive immune response, inclusively their impacts on the immune system’s response to *L. donovani*.

## Graphical Abstract

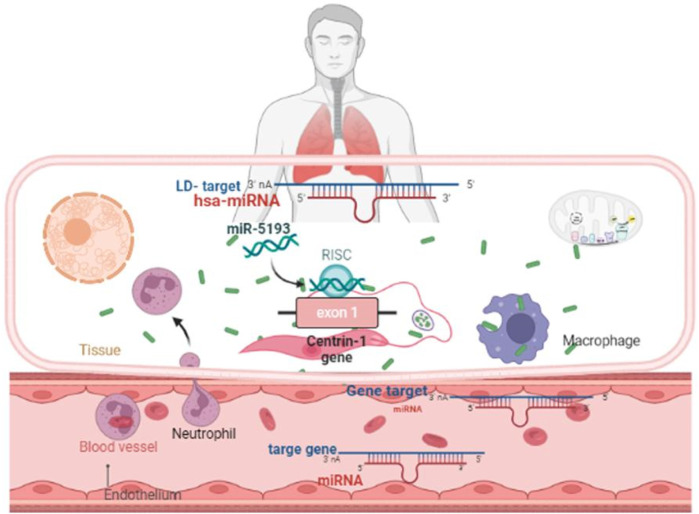

## Introduction

Leishmaniasis is considered a neglected tropical illness or disease (NTD) caused by *Leishmania* infection through sand flies (Burza et al., 2018). More than 1.7 million fresh cases of illness were reported in 98 countries each year in tropical and subtropical regions, affecting ∼1 billion individuals at risk of leishmaniasis worldwide (Alvar et al., 2012). The pathogenesis of the disease can range from confined skin ulcers called cutaneous leishmaniasis (CL) to a deadly systemic condition called visceral leishmaniasis (VL), depending on the species of the infecting *Leishmania* parasite (McCall et al., 2013; Burza et al., 2018). There are insufficient surveillance and limited treatment options for both VL and CL in the countries with the highest endemicity levels (Matlashewski et al., 2014). A preventative vaccination would be a successful strategy for protecting against this illness, lowering transmission and assisting in the global eradication of leishmaniasis. There are currently no vaccines accessible.

Centrins are a family of calcium-binding phosphoproteins localized in the centrosome that is required for centriole duplication. They may also influence cell division by severing microtubules through a calcium-mediated contraction. The human centrin genes are CETN1 (alias CEN1 or CETN), CETN2 (alias CEN2 or CALT), and CETN3 (alias CEN3 or CDC31) located at chromosomes 18, X, and 5, respectively. Moreover, in the recent past, the centrin isotypes 1–3 of protozoan parasites, for example, *Leishmania donovani* and *Trypanosoma brucei*, participate in cell division (Vats et al., 2023). Similarly, *L. donovani* has a conserved centrin-binding protein Sfi1p (LdPOC) gene like the human protein of the centriole 5 (hPOC5) gene. The human genome is abundant in protein phosphatase with EF-hand domain genes (PP7, PPEF, PPP7C, and PPP7CA), DNA damage recognition, and repair factor genes (RAD4, XP3C, p125, and XPC), whereas *L. donovani* is less defined (Korrodi-Gregório et al., 2014). Interestingly, putative centrin isotypes 4 and 5 are unique to such parasites and are not well characterized in the recent past.

Mature microRNAs (miRNAs) are 20–25 nt long, single-stranded, short noncoding RNAs that function as the powerful post-transcriptional regulators of genes. The levels of several miRNAs have been found modulated during the initial stage of the inflammatory reaction during leishmanial phagocytosis and in the late phase of the development of an adaptive immune response. miRNA molecules are functionally capable of significant tuning of vital biological processes, including inflammation and immune-responsive genes. The miRBase miRNA registry (version 22.1), which contains more than 2,000 mature human miRNAs, shows that these miRNAs are variably expressed at different times and in distinct tissue types during developmental stages. The timing of development, cell differentiation, proliferation, cell death, immune cells’ responsive genes, the modulation of intracellular signaling pathways, and the production of inflammatory mediators are all controlled by miRNAs (Bartel, 2004; Chen, 2005; Sonkoly et al., 2008). According to studies, an estimated 30% of all human genes are controlled by conserved human miRNA families (Lewis et al., 2005). According to several sources of evidence, a single miRNA can regulate 100–200 gene transcripts (RNAs) at one to several regulatory sites, or various components inside a particular biological activity that suppress an entire signaling cascade (Bartel, 2004; Grimson et al., 2007; Peter, 2010).

To ensure the effectiveness of miRNA-directed interventions, extensive and robust bioinformatics analysis is crucial beforehand, as miRNA, despite its minuscule size, plays a significant role in regulating gene expression with multiple-fold changes. The compatibility level of miRNA-binding sites during the host–pathogen transcriptome interaction requires careful consideration. Although several precise and delicate miRNA target prediction algorithms are existing, such as miRDB (Wong and Wang, 2015), miRanda (John et al., 2004), TargetScan (Agarwal et al., 2015), miRNA22 (Miranda et al., 2006), RNAhybrid (Rehmsmrier et al., 2004), and PicTar (Krek et al., 2005; Chen and Rajewsky, 2006), the high false-positive and off-target effects should be properly addressed.

The complexity of miRNA target prediction resulting from the binding and regulation of non-target genes is caused by the off-target effect. Meanwhile, GO terms and KEGG pathway enrichment analysis’s Log10 *p*-value (>0.0001) were used to screen the statistically most significant miRNA targets, and also to deduce the chances of false prediction of endogenous gene regulation. The biological processes primarily involved in the innate immune response (IIR), antigen processing and presentation (APP), mRNA 3′-terminal processing, and nuclear-transcribed messenger-RNA catabolic operation are revealed by the enrichment of GO terminology and KEGG analysis to be miRNA target genes. To attain success, it may be crucial to concentrate on delivering therapeutic microRNAs to the specific organ while minimizing any adverse effects on other targets. Despite the remarkably effective and specific computational prediction, the chance of success of RNA silencing of the desired gene *in vitro* or *in vivo* is a big challenge.

This analysis provides a contrasting difference in the number of miRNA target sites and types of miRNA seeds to deduce a theoretical perspective of the regulatory mechanism of centrin genes in *L. donovani* by host miRNAs*.* Furthermore, the interaction of centrin-binding protein and centriolar protein with centrin proteins has been assessed by *in silico* approaches employing the STRING database.

Considering the capacity of miRNAs to target-oriented genes, and *vice versa*, that is, multiple genes being battered by a single miRNA, it is necessary to perform arduous *in silico* to categorize the exact miRNA precise target sites, which must be validated through *in vitro* and *in vivo* experiments. In this study, we conducted *in silico* analysis of non-redundant mature human miRNAs that target the five isotypes of centrin genes in parasite *L. donovani*. Thus, shortlisting the few most potential miRNAs from a pool of more than 2,000 mature human miRNAs targeting the centrin-1 to centrin-5 genes would accelerate the validation and establishment of therapeutics and biomarkers for leishmaniasis.

## Materials and methods

### Gene sequence retrieval, mature miRNA sequences, and target prediction algorithms

In this study, the centrin gene reference sequences of the protozoan parasite *Leishmania donovani* were meticulously retrieved from the National Center for Biotechnology Information (NCBI) database, a reputable resource for biological information (https://www.ncbi.nlm.nih.gov/). The specific accession numbers for the centrin genes were as follows: LDBPK_221260 for centrin-1, LDBPK_366370 for centrin-2, LDBPK_342160 for centrin-3, NC_018259.1 for centrin-4, and NC_018236.1 for centrin-5. These accession numbers serve as unique identifiers for the genetic sequences under investigation. To complement the investigation of centrin genes, the study incorporated data from the miRBase microRNA repository, which is available at http://www.mirbase.org/. MiRBase hosts a comprehensive collection of microRNA sequences, encompassing a total of 2,635 distinct miRNA sequences. It is noteworthy that this repository is a subset of the 2,654 mature human miRNA sequences presently acknowledged, indicating the richness and diversity of microRNA information available for analysis. In the pursuit of identifying potential interactions between microRNAs and centrin genes, three reliable target prediction algorithms were employed. Among these, miRDB, which is a well-established and widely used tool for microRNA target prediction, played a central role. The utilization of multiple algorithms enhances the robustness and reliability of the findings, contributing to a more comprehensive understanding of the potential regulatory relationships between microRNAs and centrin genes in *Leishmania donovani*. This integrated approach underscores the significance of cross-disciplinary methodologies in unraveling the intricate molecular mechanisms underlying gene regulation in protozoan parasites. In this study, three reliable target prediction algorithms, namely, miRDB (Chen, 2005), RNA22 (Miranda et al., 2006), and RNAhybrid (Rehmsmeier et al., 2004), were used.

### *In silico* characterization of Ld-centrin 1–5 genes and encoded proteins

The centrin family of proteins, which comprises five members, include centrin 1–5. This study attempts to investigate the sequence, structure, and phylogenetic relationship among centrin 1–5, as well as host miRNAs which potentially bind and regulate their expression in *Leishmania donovani*. The Ld-centrin 1–5 genes and encoded proteins’ biochemical properties, such as their molecular weight (MW), instability index (II), aliphatic coefficient (AC), isoelectric point (pI), and grand average hydropathy (GRAVY), were calculated using the Expasy ProtParam tool (http://web.expasy.org/protparam) (Wilkins et al., 1999; Walker, 2005).

### AlphaFold-based modeling of centrin proteins and validation

AlphaFold is a unique machine learning technique that uses multi-sequence alignments to construct a deep learning algorithm while using physical and biological knowledge about the protein structure. We have predicted the structures of Ld-centrin 1–5 proteins using the AlphaFold version 2.0 pipeline based on homology modeling which predicts the secondary structure using an artificial intelligence-based algorithm. Furthermore, these generated models were cross-validated through Robetta programs. The PROCHECK server was used to construct a Ramachandran plot that evaluated and validated the modeled 3D structure. Using the Chimera v1.13.1 program, the optimal energy of predicted structures was calculated, and the template was superimposed to determine the best model with the lowest root mean square deviation (RMSD).

### Determination miRNA-binding sites in *Leishmania donovani* centrin genes

Leveraging miRDB v6.0 (http://mirdb.org/custom.html), which extracts miRNA data from miRBase v22.1 and employs the MirTarget bioinformatics tool, we applied a machine learning technique to predict the locations of miRNA targets within the provided sequences. A threshold value of 50 was applied to the anticipated target sites’ miRDB target scores to filter them. The target prediction algorithm version 2.0 of RNA22 (https://cm.jefferson.edu/rna22/Interactive/) is statistically more powerful; it uses pattern recognition to identify binding sites with a minimum of 12 base pairings within miRNA–mRNA duplexes, a seed or nucleus size of 8, the highest of two unpaired nucleotides in the seed, the allowance of G:U nucleotide wobbles in the seed zone, and a default minimum free energy (MFE). Following the energetically most advantageous miRNA hybridizations to target RNAs, dynamic programming (DP)-based RNAhybrid (https://bibiserv.cebitec.uni-bielefeld.de/rnahybrid/) forecasts single or several possible binding sites (Rehmsmeier et al., 2004).

### Endogenous human miRNA targets centered on robust and weak dataset

To ensure the accuracy and reliability of our results, we selected endogenous genes that were targets of human miRNAs with either strong or weak experimental evidence from the trustworthy and publicly accessible database, miRTargetLink (https://ccb-web.cs.uni-saarland.de/mirtargetlink/network.php). In this investigation, four key terms were employed: “*Homo sapiens*,” “microRNA name,” “target gene,” and “target pathway.” The miRTargetLink tool (version 2.0) incorporates miRNA targets, encompassing both validated targets from miRTarBase and predicted targets from mirDIP and miRDB, spanning *Rattus norvegicus*, *Mus musculus*, and *H. sapiens*.

### Target networking of miRNA gene

To visualize the target network of the miRNA gene in a concentric format, miRTargetLink2.0 was used. A one-way approach was carried out, with an emphasis on miRNAs (blue nodes) having high validation evidence (green nodes). Predicted miRNA targets that lacked validation were discarded. Enrichment analysis was also performed, with miEAA 2.0 for miRNAs and GeneTrail 3.0 for targets, to enrich once for miRNAs and twice for gene targets.

### STRING analysis for a gene network

To obtain the Ensembl IDs of genes targeted by 33 miRNAs with strong experimental evidence, a study was conducted on an online server: https://www.ensembl.org/Human/Search (Ensembl genome browser) for all genes targeted by these miRNAs. The genes obtained from Ensembl IDs were then examined for gene network via the STRING database (version 11.5) as instructed by the manufacturers with default parameters. Then, the STRING database clustered these genes into three clusters based on their gene–gene interaction networks using the K-means clustering method. The line’s color signifies the category of interaction and thickness, indicating the level of fitting in the model. Dotted lines denote the edges between clusters, with network nodes representing genes or proteins. The edges characterize gene–gene or protein–protein connotations, with magenta edges representing experimentally determined gene–gene interactions (GGIs) and purple edges signifying notorious interactions from curated databases, whereas gene interactions for neighborhood and red edges revealed the gene fusions with co-occurrence by blue color, respectively.

### GO annotation and analysis of KEGG pathway enrichment

miRNA-controlling human genes are characterized by GO annotation, and KEGG pathways are supported by mirPath v3.0 DIANA Tools (https://dianalab.e-ce.uth.gr/html/mirpathv3/index.php?r=mirpath) (Vlachos et al., 2015). Three GO categories are accessible in the GO databases (http://www.geneontology.org/, https://www.ebi.ac.uk/QuickGO/), namely, molecular function (MF), cellular component (CC), and biological process (BP), which were used for the classification of targeted genes. Defense-related genes or genes involved in vital metabolic pathways that were uniformly targeted were discovered using KEGG enrichment analysis. With a log10 *p*-value >0.0001, the statistically relevant KEGG pathways were enriched and shortlisted. Furthermore, datasets from the Database for Annotation, Visualization, and Integrated Discovery (DAVID) (https://david.ncifcrf.gov/) were enriched.

For the analysis of GO and KEGG pathways and the enrichment of particular miRNA target genes, the 0.05 *p*-value criterion, the 0.8-microT threshold, and the Fisher’s exact test enrichment analysis method (hypergeometric distribution) were all used. To mathematically validate these predicted GO terms and KEGG pathways (https://www.genome.jp/kegg/pathway.html), considerable *p*-values on the -log10P scale were converted, with log10 *p*-values greater than 0.0001 considered statistically relevant. The GO keywords’ accurate quantitative significance was determined using false discovery rate (FDR) correction and conservative statistics.

### Analysis of metabolic pathway enrichment and gene networking

DAVID version 6.8, a functional enrichment tool, had been applied to analyze the true relationship between the human genes targeted by the 33 miRNAs using solid experimental evidence. In this case, we uploaded all the genes with strong experimental support (Entrez Gene ID) toward the DAVID 6.8 gene list and used the accession conversion tool to convert them to DAVID Gene ID. Then, we utilized the default parameters for disease, functional annotation, Gene Ontology, general annotations, interactions, pathways, and protein domains in DAVID to perform the functional annotation of the genes. To accomplish clustering with high classification stringency, we used the default values for Kappa similarity, classification, enrichment thresholds, Bonferroni, and Benjamini scores.

## Results

### Sequence, structural, and phylogenetic analyses of centrin genes and encoded proteins

**FIGURE 1:**
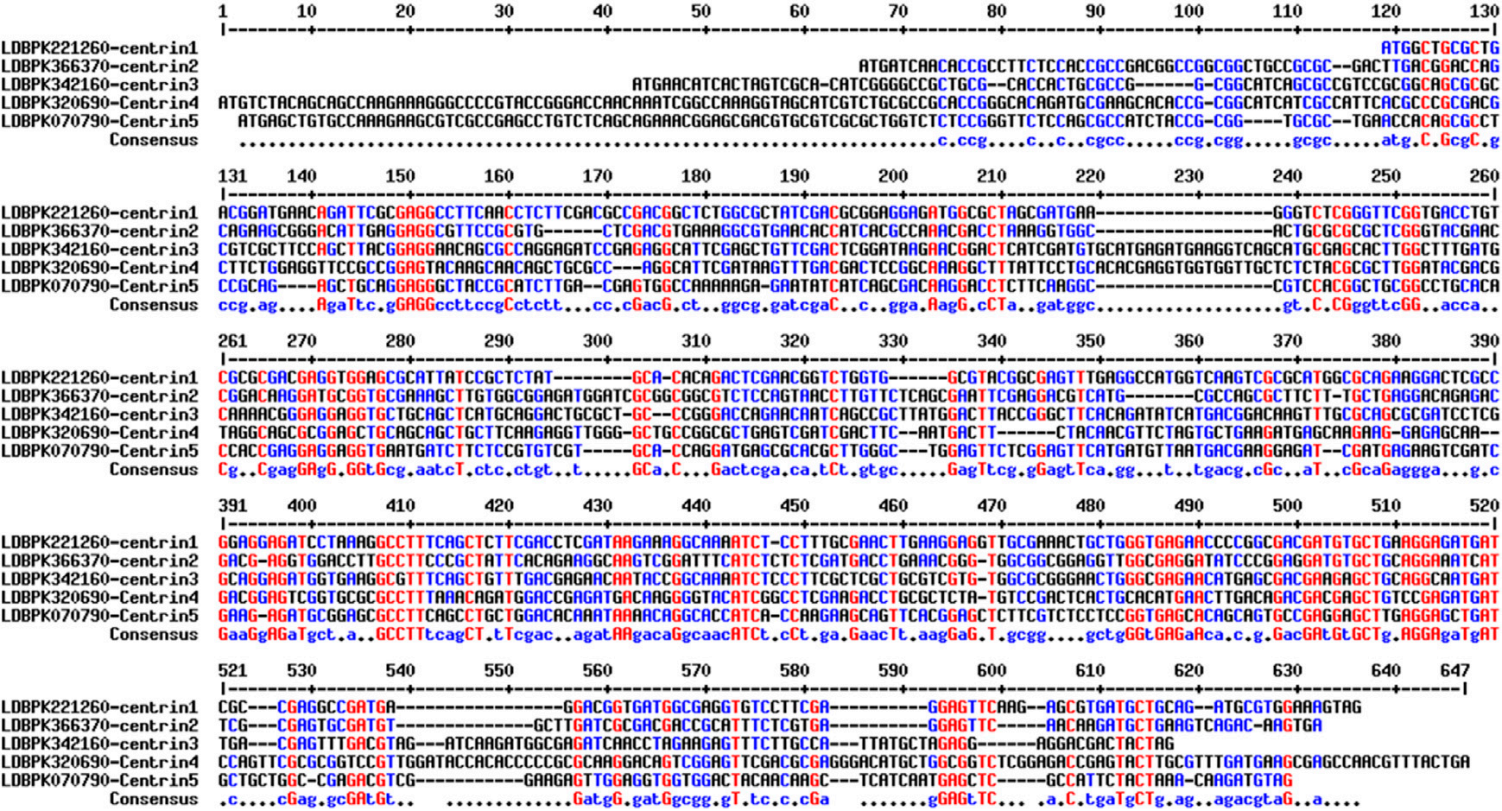
Multiple sequence alignments of centrin 1–5 nucleotide sequences. The most conserved residues are highlighted in red, less conserved residues are highlighted in blue, and least conserved nucleotides are highlighted in black.

Sequence, structural, and phylogenetic analyses of the resulting protein sequence were conducted *in silico*. Multiple sequence alignments of centrin 1–5 nucleotide sequences based on MultAlign (http://multalign.toulouse.inra.fr/multalin/) showing most conserved residues displayed in red, less conserved residues displayed in blue, and least conserved residues displayed in black (Figure 1).

The biochemical properties such as MW, PI, II, AI, and GRAVY of the Ld-centrin 1–5 encoded proteins showed that centrin 4 is the largest (22.960 KD) and centrin-1 is the smallest (16.448) protein (Table 1). According to the II value, only centrin-1 and centrin-5 are the most stable proteins. Similarly, on behalf of the Aliphatic index (AI), centrin 2 is the most aliphatic (88.00), whereas centrin 4 is the least aliphatic (71.96) (Table 1).

**TABLE 1:** Biochemical properties—MW, PI, II, AI, and GRAVY of the Ld-centrin 1–5 proteins.

Name of the centrin protein of *L. donovani* (strain BPK282A1)	UniProtKB entry	Number of amino acid (length)	Number of negatively charged residue (Asp + Glu)^a^	Number of positively charged residue (Arg + Lys)^a^	Theoretical molecular weight (MW) in KD^a^	Theoretical isoelectric point (pI)^a^	Instability index (II)^a^	Aliphatic index (AI)^a^	Grand average of hydropathicity (GRAVY)^a^	Protein structure prediction	Domain (D) and disordered region (DR) with an amino acid position
Centrin-1	E9BFZ1	149	33	18	16.448	4.41	30.59	79.33	−0.360	AlphaFold	D1 (4–39)
											D2 (78–113)
											D3 (114–149)
Centrin-2	E9BUZ4	165	40	26	18.899	4.50	40.12	88.00	−0.573	AlphaFold	D1 (21–56)
											D2 (1310165)
Centrin-3	E9BR14	181	38	22	20.784	4.46	59.51	72.87	−0.675	AlphaFold	DR (1–24)
											D1 (34–69)
											D2 (110–145)
											D3 (146–181)
Centrin-4	E9BNH3	209	30	24	22.960	5.28	52.29	71.96	−0.433	AlphaFold	DR (1–20)
											D1 (50–85)
											D2 (123–158)
Centrin-5	E9B970	187	34	21	20.620	4.58	37.78	77.22	−0.463	AlphaFold	DR (1–21)
											D1 (25–60)
											D2 (116–151)
											D3 (152–187)

^a^ These parameters are calculated using the ExPASy, ProtParam tool.

*Instability index (II) classifies the protein as either stable or unstable (II < 40 means the protein is stable). Because none of these proteins includes any tryptophan (Trp) residues, the calculated extinction coefficient may be erroneous by more than 10%.

**FIGURE 2:**
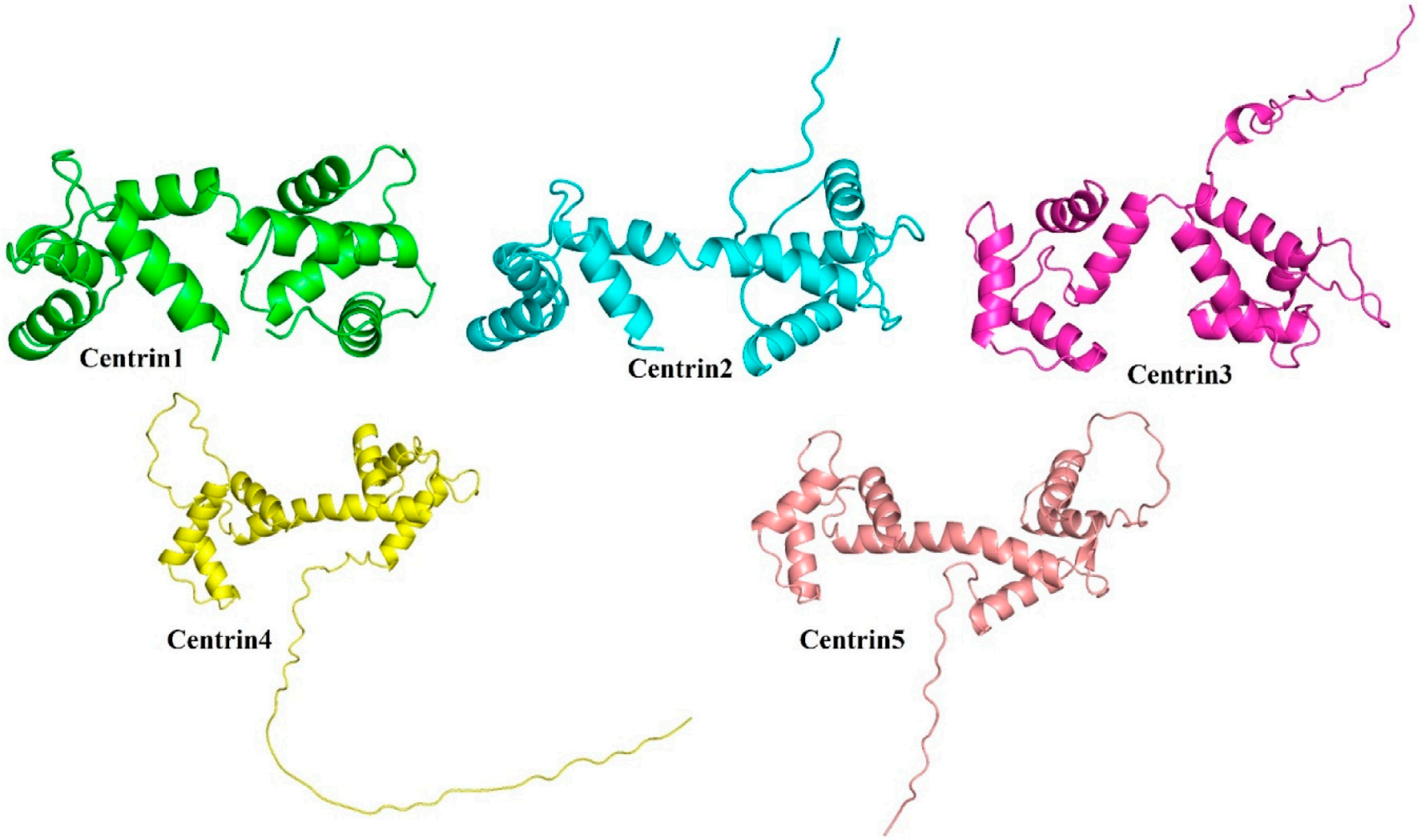
AlphaFold-based modeling and structure prediction of centrin proteins of *L. donovani*.

**FIGURE 3:**
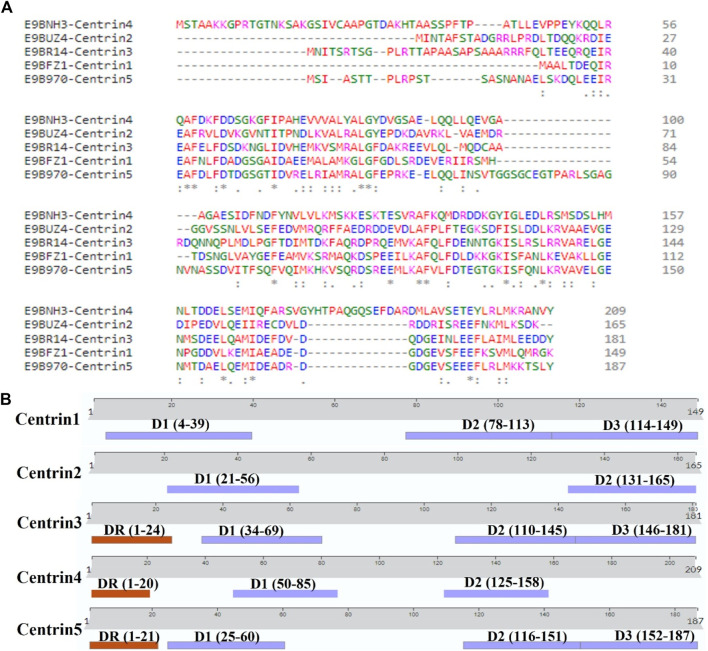
**(A)** CLUSTAL Omega version 1.2.4 showing multiple sequence alignments of centrin 1–5 amino acid sequences and **(B)** *L. donovani* centrin 1–5 proteins showing the position of conserved domains and disordered regions.

The AlphaFold (version 2.0)-based homology modeling of the predicted secondary structure of centrin 1–5 shows that these proteins have only alpha-helical secondary elements and some disordered regions (Figure 2). These disordered regions (DRs) and highly conserved domains (D) are located in different regions of centrin proteins, which was confirmed by the CLUSTAL Omega alignment program (Figure 3A), and they are represented by a schematic diagram (Figure 3B). Centrin 3–5 have a single DR, whereas DR is completely absent in centrins 1 and 2. Interestingly, centrins 1, 3, and 5 have three major conserved domains, namely, D1, D2, and D3, whereas centrins 2 and 4 proteins have only two conserved domains, namely, D1 and D2 (Figure 3B).

### Analysis of human miRNAs targeting centrin genes of *L. donovani*

**FIGURE 4:**
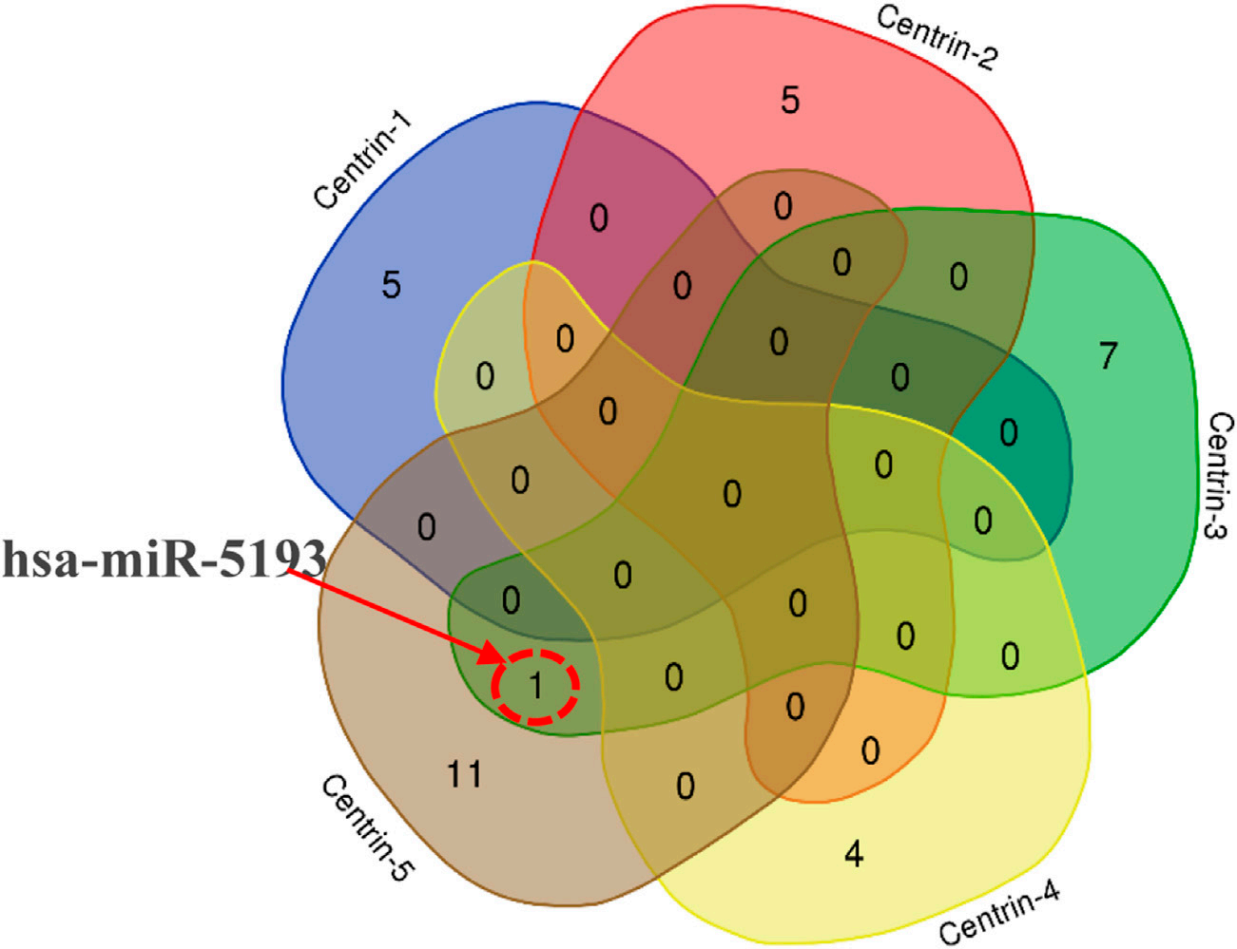
Intersection of miRNA targeting all five centrin-1 to centrin-5 gene isotypes is depicted by a Venn diagram. hsa-miR-5193 was the only human miRNA found unanimously targeting centrin-3 and centrin-5 genes.

In this study, the miRDB server was employed to identify such human miRNAs which have potential to target the centrin 1–5 genes of *L*. *donovani* and efficiently regulate RNA silencing. In this computational analysis, it was found that the centrin-3 (accession no. LDBPK_342160) and putative centrin-5 (accession no. NC_018236.1) genes of L. *donovani* were targeted by eight and twelve out of 2,635 known human miRNAs. hsa-miR-5193 was the only human miRNA found unanimously targeting centrin-3 and centrin-5 genes (Figure 4; Table 2), whereas centrin-1 (LDBPK_221260), centrin-2 (LDBPK_366370), and putative centrin-4 (NC_018259.1) were having four to five regulatory sites from a pool of human miRNAs (Table 2).

**TABLE 2:** Centrin gene isotypes 1–5 of parasite *Leishmania donovani* were targeted by only 33 out of 2,635 nonredundant mature human miRNAs above the threshold parameters of an miRDB target prediction algorithm.

Accession no. of TriTrypDB or NCBI GenBank	Names of the gene	Location on chromosome no.	Gene length (no. of nucleotides)	No. of targeting human miRNA	Human miRNAs targeting the centrin 1–5 genes
LDBPK_221260	Centrin-1	22	450	5	hsa-miR-6784-3p, hsa-miR-3142, hsa-miR-1245b-5p, hsa-miR-205-5p, and hsa-miR-1184
LDBPK_366370	Centrin-2	36	498	5	hsa-miR-11399, hsa-miR-431-3p, hsa-miR-3692-5p, hsa-miR-4516, and hsa-miR-4297
LDBPK_342160	Centrin-3	34	546	8	hsa-miR-5193, hsa-miR-5197-5p, hsa-miR-1976, hsa-miR-6894-3p, hsa-miR-7156-5p, hsa-miR-4273, hsa-miR-4660, and hsa-miR-3680-3p
LDBPK_320690 (NC_018259.1)	Centrin-4	32	630	4	hsa-miR-4785, hsa-miR-549a-5p, hsa-miR-383-3p, and hsa-miR-6717-5p
LDBPK_070790 (NC_018236.1)	Centrin-5	9	561	12	hsa-miR-6071, hsa-miR-4753-3p, hsa-miR-6736-3p, hsa-miR-5193, hsa-miR-1236-3p, hsa-miR-4530, hsa-miR-103a-2-5p, hsa-miR-103a-1-5p, hsa-miR-588, hsa-miR-4701-5p, hsa-miR-556-5p, and hsa-miR-3682-3p

### Identification of miRNA-binding sites

**FIGURE 5:**
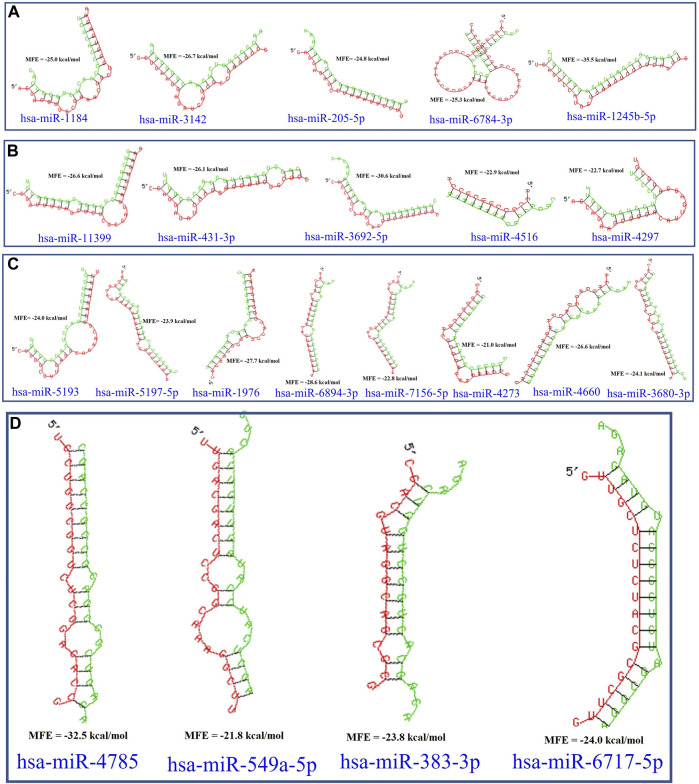
*L. donovani*’s **(A)** centrin-1 gene is targeted by five different miRNAs. **(B)** Centrin-2 gene is also accessed by five different human miRNAs. **(C)** Eight different miRNAs are targeting centrin-3 gene. **(D)** Four human miRNAs are regulating the centrin-4 gene of *L. donovani*. These duplex structures of miRNAs (green) and their mRNA targets (red) were generated using the RNAhybrid tool.

In this study, the initial attempt was made to identify cellular miRNAs with targets exceeding 50 and mean free energy below -20 kcal/mol (Tables 3–7) that target all five reference centrin 1–5 genes. To complement and strengthen the results of the miRDB algorithm, these miRNAs were re-accessed using RNA22 and RNAhybrid programs. Meanwhile, the RNA22 program generates energetically optimum hairpin structures to show the binding sites of human miRNAs in centrin 1–5 mRNA genes in *Leishmania donovani*. An energetically most favorable hairpin and loop are shown for the representative miRNAs (green) and their mRNA target duplexes (red) (Figures 5A–D). Among centrin genes 1–5, the MFE (>-20 kcal/mol) of miRNA (green) and their mRNA target (red) duplex topologies differ (Figures 5A–D). hsa-miR-1184 targets the centrin-1 gene of *L. donovani* with the strongest interaction because it forms the energetically most stable complex with the least MFE of −35.50 kcal/mol (Table 3; Figure 5A). Similarly, hsa-miR-3692-5p, hsa-miR-6894-3p, and hsa-miR-4785 are forming a energetically more stable complex with the centrin-2, centrin-3, and centrin-4 genes of *L. donovani* (Tables 4–6; Figures 5B–D).

**TABLE 3:** Centrin-1 gene targeted by five human miRNAs having an miRDB target score (>50) and MFE (>-20 kcal/mol). The rightmost column is showing miRNA-target heteroduplex in the centrin-1 gene as drawn using the RNA22 algorithm.

Human miRNA name	miRDB target score	MFE energy (Kcal/mol)	Target position (left most) in the centrin-1 gene	miRNA-target heteroduplex
hsa-miR-6784-3p	58	−25.00	311	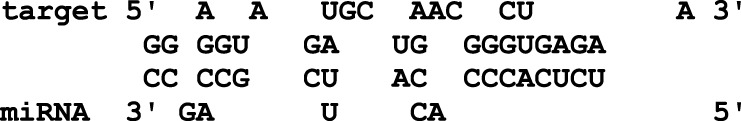
hsa-miR-3142	58	−26.70	354	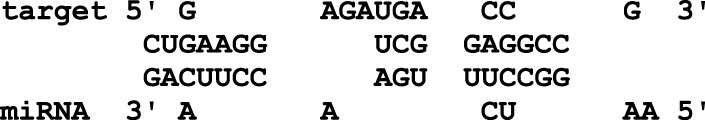
hsa-miR-1245b-5p	58	−24.80	241	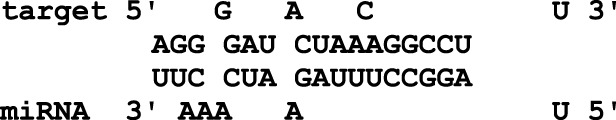
hsa-miR-205-5p	53	−25.30	57	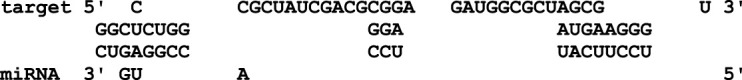
hsa-miR-1184	52	−35.50	198	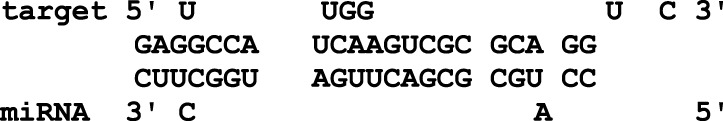

**TABLE 4:** The centrin-2 gene targeted by five human miRNAs having miRDB target score (≥50) and the MFE (>−20 Kcal/mol). The rightmost column is showing miRNA-Target heteroduplex in centrin-2 gene as drawn by RNA22 algorithm.

Human miRNA name	miRDB target score	MFE energy (Kcal/mol)	Target position (left most) in the centrin-2 gene	miRNA-target heteroduplex
hsa-miR-11399	76	−26.60	335	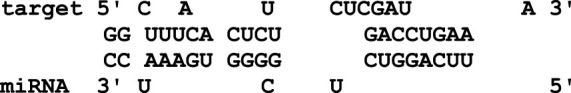
hsa-miR-431-3p	71	−26.10	384	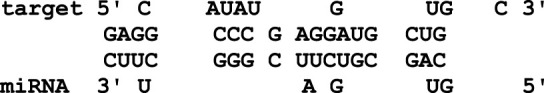
hsa-miR-3692-5p	53	−30.60	18	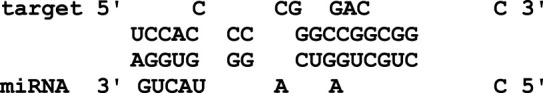
hsa-miR-4516	51	−22.90	10	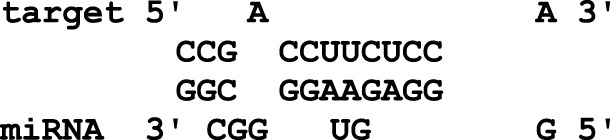
hsa-miR-4297	50	−22.70	62	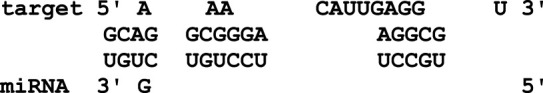

**TABLE 5:** The centrin-3 gene targeted by eight human miRNAs having miRDB target score (≥50) and the MFE (>−20 Kcal/mol). The rightmost column is showing miRNA-Target heteroduplex at the left most position of centrin-3 gene sequence.

Human miRNA name	miRDB target score	MFE energy (Kcal/mol)	Target position (left most) in the centrin-3 gene	miRNA-target heteroduplex
hsa-miR-5193	80	−24.00	196	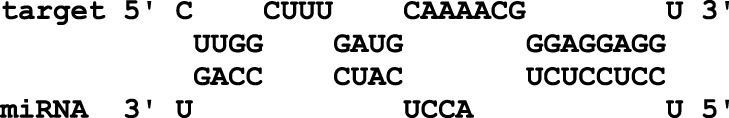
hsa-miR-5197-5p	79	−23.90	496	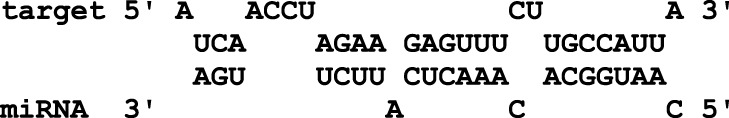
hsa-miR-1976	71	−27.70	320	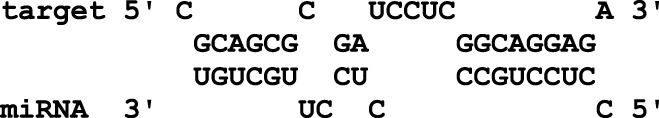
hsa-miR-6894-3p	69	−28.60	439	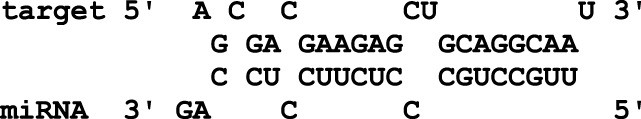
hsa-miR-7156-5p	68	−22.80	353	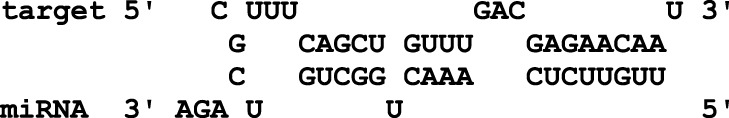
hsa-miR-4273	61	−21.00	132	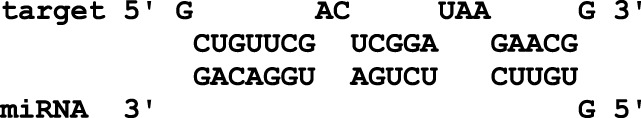
hsa-miR-4660	57	−26.60	434	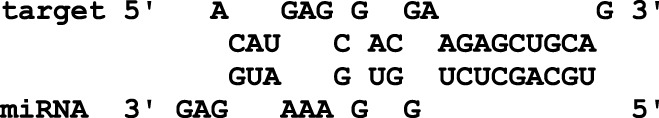
hsa-miR-3680-3p	53	−24.10	392	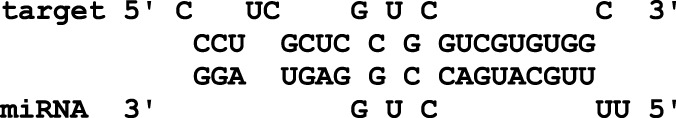

**TABLE 6:** The centrin-4 gene targeted by four human miRNAs having miRDB target score (≥50) and the MFE (>−20 Kcal/mol). The rightmost column is showing miRNA-Target heteroduplex at the left most position of centrin-4 gene sequence.

Human miRNA name	miRDB target score	MFE energy (Kcal/mol)	Target position (leftmost) in the centrin-4 gene	miRNA-target heteroduplex
hsa-miR-4785	70	−32.50	572	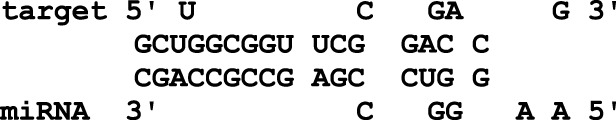
hsa-miR-549a-5p	61	−21.80	185	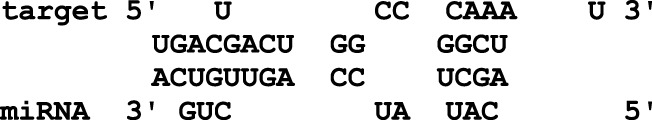
hsa-miR-383-3p	57	−23.80	252	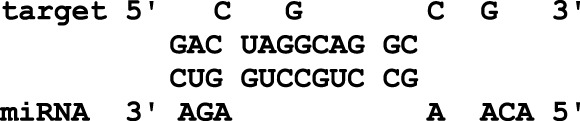
hsa-miR-6717-5p	53	−24.00	229	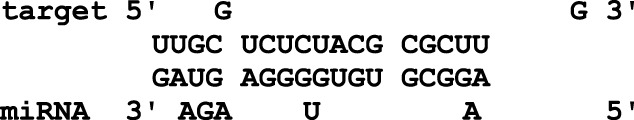

**TABLE 7:** The centrin-5 gene targeted by twelve human miRNAs having miRDB target score (>50) and the MFE (>−20 Kcal/mol). The rightmost column is showing miRNA-Target heteroduplex at the left most position of centrin-5 gene sequence.

Human miRNA name	miRDB target score	MFE energy (Kcal/mol)	Target position (leftmost) in the centrin-5 gene	miRNA-target heteroduplex
hsa-miR-6071	71	−25.80	23	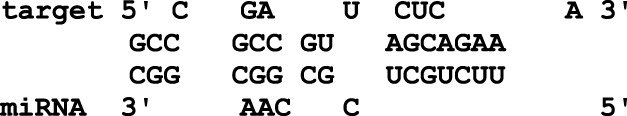
hsa-miR-4753-3p	65	−25.30	277	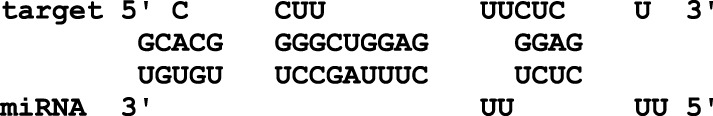
hsa-miR-6736-3p	61	−27.90	448	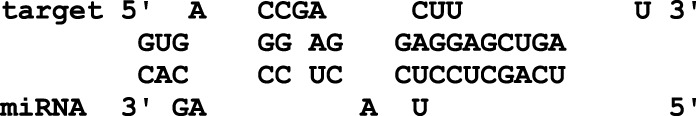
hsa-miR-5193	59	−26.20	476	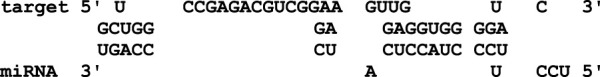
hsa-miR-1236-3p	57	−28.00	477	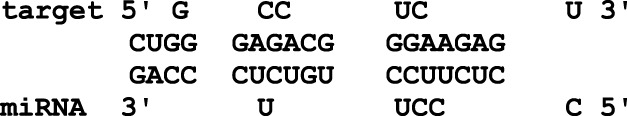
hsa-miR-4530	57	−27.90	358	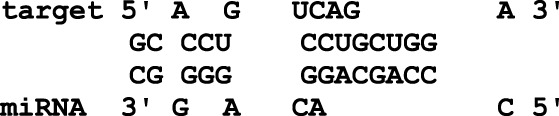
hsa-miR-103a-2-5p	56	−26.40	438	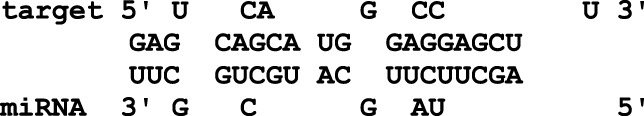
hsa-miR-103a-1-5p	56	−26.40	438	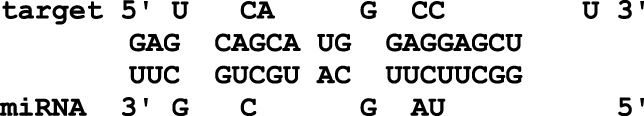
hsa-miR-588	54	−22.50	137	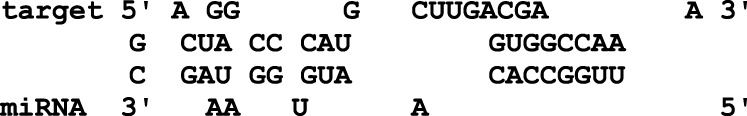
hsa-miR-4701-5p	54	−24.10	491	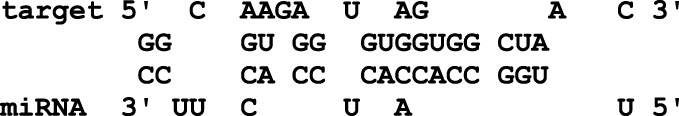
hsa-miR-556-5p	54	−25.10	522	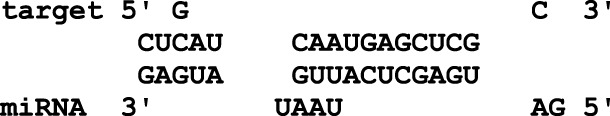
hsa-miR-3682-3p	53	−23.00	23	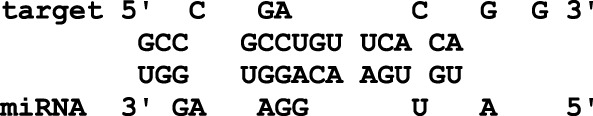

### Human miRNAs determined endogenous targets depending on the strong and weak experimental signal

**FIGURE 6:**
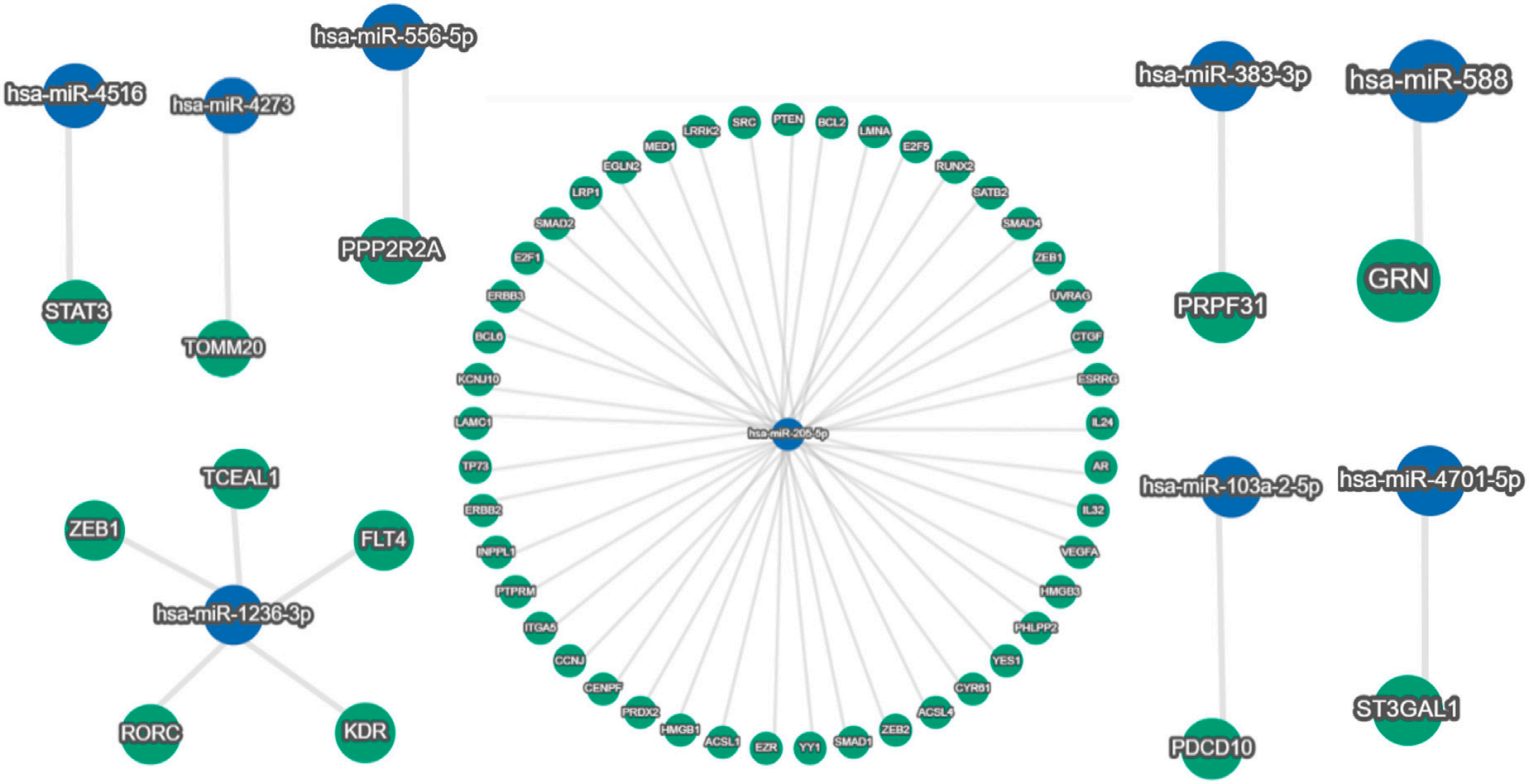
Around 490 human genes were found targeted by hsa-miR-205-5p (blue node in the center), out of which 136 gene-miR-205-5p interactions have strong (green nodes) validation evidence. In addition, hsa-miR-4516, hsa-miR-4273, hsa-miR-383-3p, hsa-miR-103a-2-5p, hsa-miR-588, hsa-miR-4701-5p, and hsa-miR-556-5p are showing single gene targets with strong experimental evidence; meanwhile, five human genes are interacting with hsa-miR-1236-3p with strong (green nodes) validation evidence (miRTargetLink version 2.0 database).

This study is set out to identify human miRNAs, comprehend their roles, and ascertain how they regulate endogenous targets. To refine the predictions, we considered miRNAs that have been experimentally validated. We chose one that had substantial experimental support. Certain human miRNAs are more prevalent than others, and they have a greater regulatory effect on a greater number of genes. After being sorted using miRTargetLink version 2.0, Table 8 shows the number of human genes targeted by host miRNAs with good and weak experimental evidence. Most remarkably, it was found that out of 33 host miRNAs, only nine miRNAs (hsa-miR-205-5p, hsa-miR-4516, hsa-miR-4273, hsa-miR-383-3p, hsa-miR-1236-3p, hsa-miR-103a-2-5p, hsa-miR-588, hsa-miR-4701-5p, and hsa-miR-556-5p) have strong experimental evidence of endogenous targets (Figure 6) (Table 8). It was observed that 136 genes were identified as the target genes of hsa-miR-205-5p, with strong experimental evidence, whereas 454 genes had weak experimental evidence (as shown in Table 8).

**TABLE 8:** Number of human genes targeted by host miRNAs with strong and weak experimental evidence as assorted from three databases (data source-miRTargetLink version 2.0).

miRNA name	No. of targeted genes with strong experimental evidence (miRTargetLink 2.0)	No. of targeted genes with weak experimental evidence (miRTargetLink 2.0)
Centrin-1		
hsa-miR-6784-3p	0	196
hsa-miR-3142	0	100
hsa-miR-1245b-5p	0	104
hsa-miR-205-5p	136	454
hsa-miR-1184	0	268
Centrin 2		
hsa-miR-11399	0	0
hsa-miR-431-3p	0	36
hsa-miR-3692-5p	0	176
hsa-miR-4516	2	526
hsa-miR-4297	0	208
Centrin 3		
hsa-miR-5193	0	656
hsa-miR-5197-5p	0	424
hsa-miR-1976	0	1,168
hsa-miR-6894-3p	0	154
hsa-miR-7156-5p	0	189
hsa-miR-4273	2	184
hsa-miR-4660	0	190
hsa-miR-3680-3p	0	478
Centrin 4		
hsa-miR-4785	0	90
hsa-miR-549a-5p	0	0
hsa-miR-383-3p	2	1,034
hsa-miR-6717-5p	0	74
Centrin 5		
hsa-miR-6071	0	154
hsa-miR-4753-3p	0	716
hsa-miR-6736-3p	0	510
hsa-miR-1236-3p	10	532
hsa-miR-4530	0	324
hsa-miR-103a-2-5p	2	172
hsa-miR-103a-1-5p	0	0
hsa-miR-588	2	518
hsa-miR-4701-5p	2	562
hsa-miR-556-5p	2	158
hsa-miR-3682-3p	0	160

### miRNA–gene target networking

After screening a total of nine miRNAs, we discovered that hsa-miR-205-5p targets 136 human genes (Figure 6). Hence, only 45% of these relationships have substantial validation data. Similar to this, there is substantial evidence that just five genes interact with hsa-miR-1236-3p. Strong experimental evidence was found for single-gene targets for the remaining seven miRNAs. Nevertheless, the miRNA–gene interaction network of those with limited experimental evidence is not displayed (data not shown) because of a huge and complex interaction network.

### Gene–miRNA interaction network regulated by nine human miRNAs based on STRING-DB analysis

**FIGURE 7:**
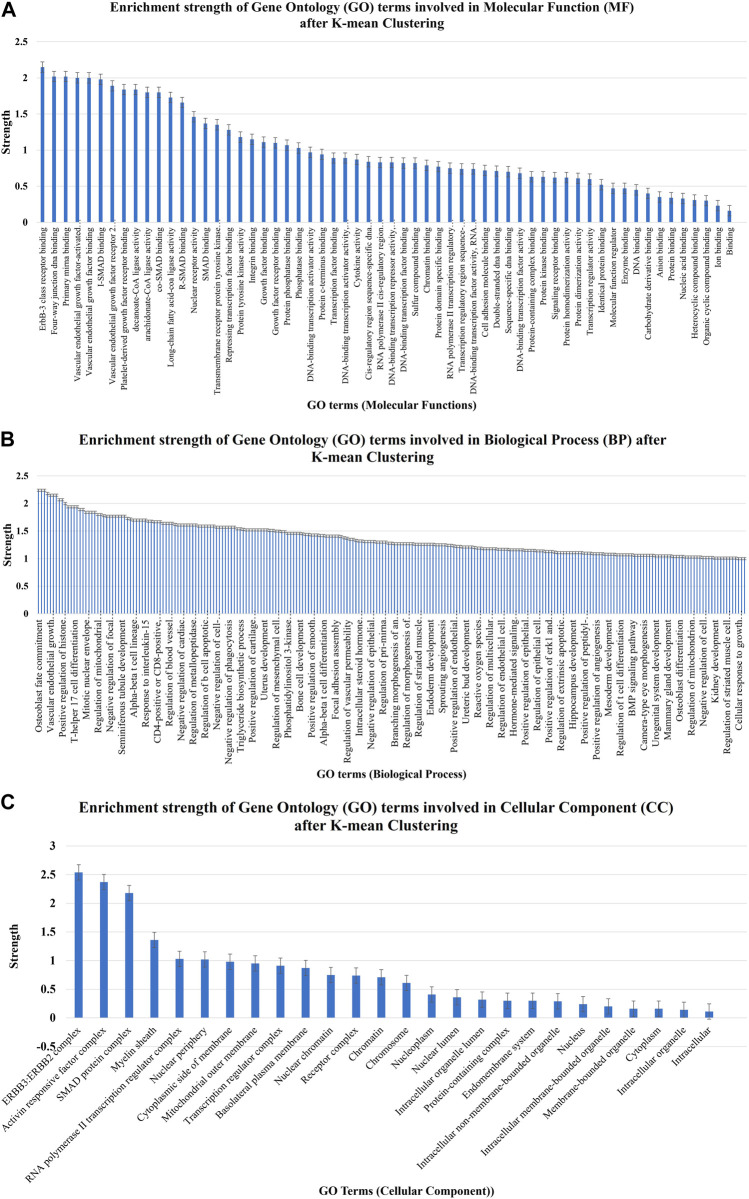
Enrichment strength of significant GO terms involved in **(A)** molecular function, **(B)** biological process, and **(C)** cellular component after K-mean clustering which were targeted by nine potential human miRNAs.

The strength enrichments of the GO term were segregated into three groups, namely, BP, CC, and MF, based on their strength and false discovery rate. The STRING analysis uncovered that 60, 83, and 26 GO terms associated with MF, BP, and CC, respectively, were found to be enriched in the strength of the gene–miRNA interaction network, as shown in Figures 7A–C. GO:0043125 for ErbB-3 class receptor binding, GO:0000400 for four-way junction DNA binding, GO:0070878 for primary miRNA binding, GO:0005021 for vascular endothelial growth factor-activated receptor activity, GO:0038085 for vascular endothelial growth factor binding, GO:0070411 for I-SMAD binding, GO:0043184 for vascular endothelial growth factor receptor 2 binding, GO:0005161 for platelet-derived growth factor receptor binding, GO:0102391 for decanoate-CoA ligase activity, and GO:0047676 for arachidonate-CoA ligase activity are the top 10 most strength enriched GO terms involved in MF after K-mean clustering (Figure 7A and Supplementary Material S1). These GO MFs are mostly associated with 16 human genes, namely, ERBB2, ERBB3, YY1, HMGB1, HMGB3, SMAD1, SMAD2, SMAD4, FLT4, KDR, STAT3, ITGA5, VEGFA, PTEN, ACSL1, and ACSL4 (Supplementary Material S1).

### Target gene analysis through KEGG pathway enrichment

**FIGURE 8:**
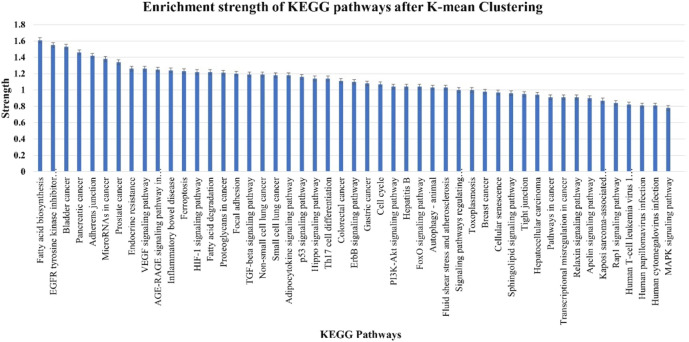
Enrichment strength of most significant KEGG pathways targeted by nine cellular miRNAs after K-mean clustering.

A total of 49 KEGG pathways that were highly statistically significant (log10*p* > 0.0001) were enhanced and pre-selected from miRTarget database Link 2.0 (Figure 8). Using such an approach, the erroneously identified KEGG pathways and their genes were reduced, and the most important genes were categorized into functional groupings. A total of 230 crucial genes were identified on 6,746 background genes from miRTarget database Link 2.0. Through intensive analysis, 34 KEGG pathways with high predictive strength (>1) and low FDR were chosen for the analysis of miRNA–gene networks. Figure 8 and Supplementary Material S2 display these KEGG pathways and the associated number of target genes.

### GGI network designated by miRNAs

**FIGURE 9:**
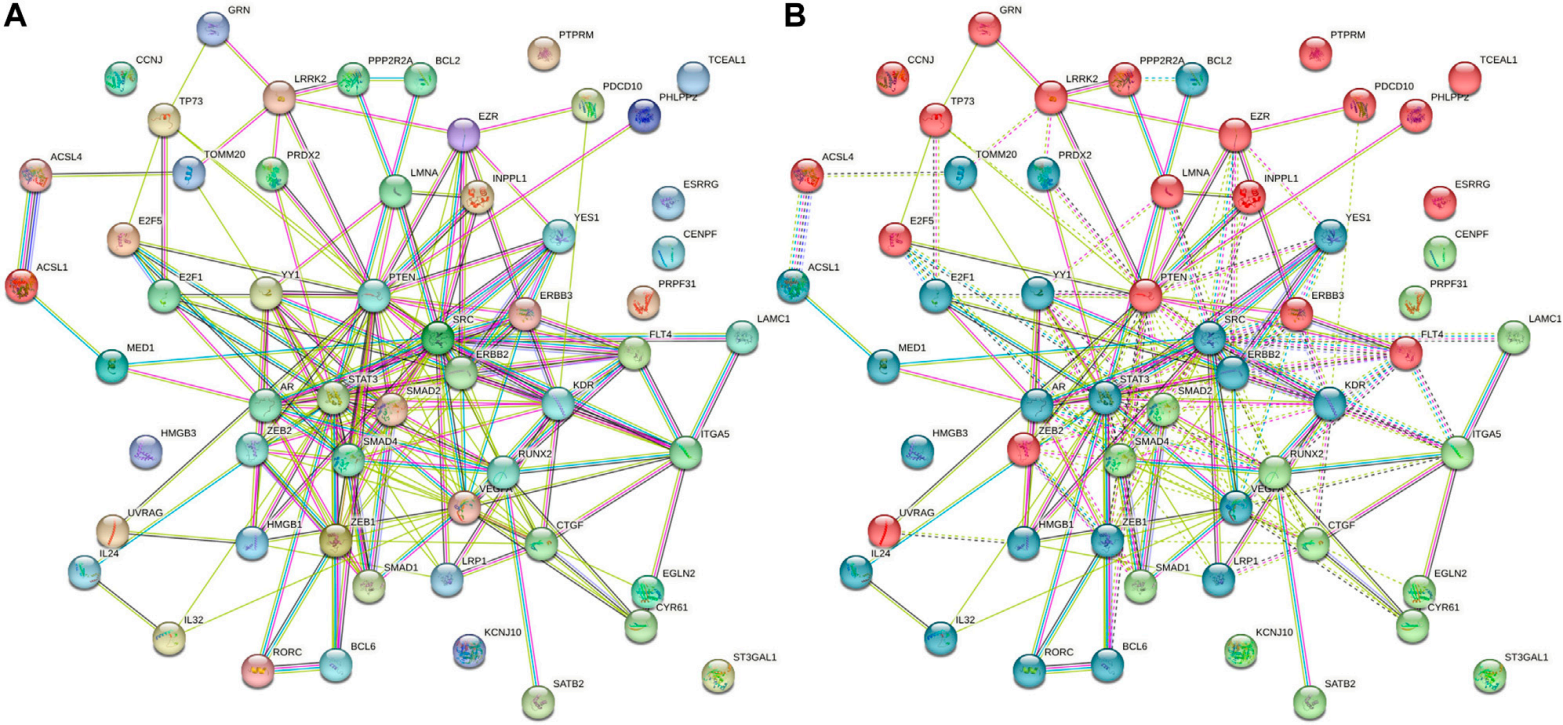
**(A)** Non-clustered gene–gene interaction network showing as it is for host (human) genes due to *L. donovani* perturbation. **(B)** K-mean enriched triclustered GGI network showing first-, second-, and third-level interactors in red (20 genes), green (14 genes), and blue (22 genes), respectively. There are 56 nodes having 184 edges with an average degree of nodes 6.57 and GGI enrichment *p*-value <1.0e-16.

Based on the degree of interactions, the K-means grouping of genes produced three clusters for the 56 genes in the GGI network. An average degree of 6.57 was found for the 56 nodes with 184 edges, according to the STRING and Cytoscape analysis. The network exhibited considerably more interconnections, showing that genes were physiologically related together, according to the GGI enrichment *p*-value of less than 1.0e–16. The red cluster of 20 genes, the green cluster of 14 genes, and the blue cluster of 22 genes represented the first, second, and third shells of interactors, respectively. This finding suggests that the GGI network has more interconnections, indicating that genes are biologically linked together. The genes, namely, *EZR*, *INPPL1*, *LMNA*, *PTEN*, *FLT4*, *ERBB3*, *ZEB1*, *ZEB2*, *YES1*, *KDR*, *SRC*, *ERBB2*, *STAT3*, *AR*, *LRP1*, *SMAD1*, *SMAD2*, *SMAD4*, *CTRF*, *RUNX2*, *VEGFA*, *YY1*, and *E2F1*, formed an intricate GGI network (red oval, Figure 9). Interestingly, it was found that the EZR, INPPL1, LMNA, PTEN, FLT4, ERBB3 and ZEB2 genes are the central regulator of the first-level interactors. Similarly, YES1, SRC, ERBB2, STAT3, AR, and KDR genes are the second-tier interactors; meanwhile, SMAD1, SMAD2, SMAD4, RUNX2, and CTGF genes are the third-tier interactors.

In the plasma membrane, the EZR gene (also known as vilin 2 or Ezrin) takes part in the connections of important cytoskeletal structures, which is essential for the development of microvilli and membrane ruffles on the apical pole in epithelial cells. It is necessary for typical micropinocytosis along with PLEKHG6 and A-kinase anchoring proteins. The nuclear lamina, which is a layer of fibers on the side of the inner nuclear membrane closest to the nucleus, is composed of either the LMNA gene (also known as prelamin-A/C) or lamin proteins. It serves as a structure for the nuclear envelope and also interacts with chromatin. Mammal lamina has similar numbers of lamin A and lamin C proteins, which are crucial for nuclear assembly, chromatin architecture, nuclear membrane, and telomere dynamics. Moreover, it is necessary for the proliferation of muscle satellite cells as well as the correct development of the skeletal muscle and peripheral nervous system. Furthermore, it is necessary for bone growth and osteoblastogenesis. The transcriptional inhibitor zinc finger E-box-binding homeobox 2 (ZEB2) binds to the DNA sequence 5′- CACCT-3′ in several promoters. E-cadherin, ZF class homeoboxes, and pseudogene transcription are repressed.

YES proto-oncogene 1 (YES1) is recruited for the phosphorylated receptor as a result of stimulation by receptor tyrosine kinases (RTKs), which include EGRF, PDGFR, CSF1R, and FGFR. This results in the activation and phosphorylation of downstream substrates. The phosphorylation of PARD3 was promoted to favor epithelial tight junction formation during EGFR activation. Transcription factor (TF) E2F5 is a transcriptional activator that binds to E2F sites in the promotion of several genes whose products contribute to cell proliferation. Growth factor-induced signal transduction may be mediated. It helps resting cells respond to growth factor stimulation early on, which is particularly essential for multiciliate cell differentiation, and helps bind and activate genes required for centriole biogenesis in collaboration with MCIDAS and E2F5.

## Discussion

Small, evolutionarily conserved regulatory noncoding RNAs, known as 22–25 nucleotide mRNAs, are believed to regulate the transcriptional and post-transcriptional gene expression in cellular signaling pathways across various eukaryotes, including humans (Felden and Gilot, 2018; Paul et al., 2018; Paul et al., 2020a; Paul et al., 2020b; Condrat et al., 2020; Baig and Krishnan, 2021). The expression of the target mRNAs is controlled by mRNA’s degradation, inhibition of translation, or both in response to the perfect or imperfect complementary base pairing of mRNAs to the target mRNAs. It is clear from several examples that mRNAs affect a wide range of biological processes in cells, such as cell proliferation and differentiation, growth and development, metabolism, cell death, signal transduction, and immune regulation (Miska, 2005; We and Lu, 2016; Pockar et al., 2019). Over 60% of human protein-coding genes are said to be regulated by mRNAs (Friedman et al., 2009).

The idea that mRNAs participate in intricate regulatory networks is supported by approximately 2,654 unique mature human miRNA sequences that are available in miRBase. It has been shown that the host mRNAs regulate cellular responses under different pathophysiological conditions, such as cancer, autoimmune diseases, infectious diseases, hereditary conditions, and metabolic disorders that change physiological and signaling processes (Garo and Murugaiyan, 2016; Bruscella et al., 2017; Zhou et al., 2017; Baig et al., 2023).

Given that parasites and their particular hosts are known to interact in complex manners, research into these interactions for infectious diseases is at the cutting edge. When mRNAs are discharged into vesicles or moving extracellular fluids termed exosomes, they have the potential to serve as biomarkers for a wide spectrum of diseases, including parasite infections. miRNAs have been connected to the leishmaniasis pathogenesis over the past 10 years. By biting infected female, phlebotomine sandflies, *Leishmania* is transmitted to vertebrates, where it causes leishmaniasis, which can range from skin lesions to lethal leishmaniasis (Akhoundi et al., 2017; Borghi et al., 2017; Derici et al., 2018; Vats et al., 2023).

It has been demonstrated that the *L. major’s* (*Leishmania major*) early hours are negatively regulated in terms of the control of the toll-like-receptor (TLR) signaling and targeting transcripts NFB p105 (NFKB1), Myd88, Interferon (TRIF), TRAF6, and IRAK1 in macrophages, which are known as the negative regulators of precisely calibrated inflammatory reactions. When let-7a, miR-25, miR-26a, miR-132, and miR-140 were overexpressed in human macrophages infected with *L. major*, it negatively correlated with the expression of their specific chemokine targets CCL2, CCL5, CXCL10, CXCL11, and CXCL12 (Guerfali et al., 2008; Bazzoni et al., 2009; Lemaire et al., 2013). The target genes CD40 and TNF receptors (TNFRs) are connected with the downregulation of miR-193b and the overexpression of miR-671, respectively, which modulate the inflammatory response in the parasite-caused lesions (Nunes et al., 2018). Lemaire et al. (2013) also emphasized the downregulation of miR-210 during *L. infantum* infection caused by procaspase-3 to be targeted in macrophages derived from monocytes, thereby inhibiting cell apoptosis (Chen and Xiaowei, 2020). Diotallevi et al. (2018) found that miR-346 was upregulated in two human cell line (U937 and THP-1)-derived macrophages after infection with four different strains or isolates of *L. infantum*. This suggests that miR-346 may play a role in the host immune response to *L. infantum* infection in macrophages. The *L. infantum*-infected human cell line-derived macrophages decreased the mRNA level of MHC or interferon (IF)-associated genes, such as regulatory factor X1 (RFX1), antigen peptide transporter 1 (TAP1), and B-cell receptor-associated protein 31 (BCAP31), which was implicated in both immune response modulation and cell survival during infection under endoplasmic reticulum stress; thus, miR-346 is assumed to have implications for the development of new therapeutic strategies for the treatment of leishmaniasis. Ghosh et al. (2013) examined an intriguing relationship between altered lipid metabolisms during *L. donovani* infection of Huh7 cell lines and found that miR-122 levels were downregulated due to the affected DICER1.

In addition, miR-21 (downregulated) and miR-146a (upregulated) have been shown to have strong correlations in *L. major* infection by Geraci et al. (2015) and Wei and Schober (2016) as miR-146a has an anti-inflammatory role. The specific TGF-signaling pathway members SMAD7 and TRAF6 were also affected by *L. Donovani*-infected dendritic cells. The upregulation of miR-30a-3p during infection has been reported to be a trigger for *L. donovani*, which is characterized by the suppression of the autophagic process in THP-1 and human monocyte-derived macrophages by the negative regulation of Beclin-1 (BECN1) (Singh et al., 2016). Autophagy is a process by which cells break down and recycle their cellular components, including invading pathogens, to maintain cellular homeostasis.

Despite studies emphasizing the importance of miRNAs as the regulators of gene expression in the pathophysiology of numerous human disorders, it is still unclear how miRNAs specifically function in human parasitic infections. Therefore, understanding the regulatory functions of miRNAs in host–parasite interactions will not only offer fresh perspectives on the pathogenesis of parasite disease but also lay the groundwork for the development of novel therapeutic strategies. In this study, we made a concerted effort to clarify the role of specific human miRNAs in controlling the *Leishmania* genes, as well as their host genes and their intricate network of interactions, to clinically understand the onset and progression of the dreaded Leishmaniasis parasitic disease in humans, and use these specific miRNAs as potential diagnostic biomarkers.

High-mobility group box proteins (HMGB1 and HMGB3) are multifunctional, redox-sensitive proteins that play a variety of roles in various cellular compartments. HMGB1 and HMGB3 genes encode these proteins which serve as ligands for TLRs (Liang and He, 2022). HMGB1, a key chromatin-associated non-histone protein in the nucleus, plays a role in DNA repair and genome stability (Chen et al., 2022) in addition to its function as a DNA chaperone during processes such as replication, transcription, chromatin remodeling, V(D)J recombination, and inflammatory response coordination (Kang et al., 2014).

The cytoplasmic HMGB proteins HMGB1, HMGB2, and HMGB3 have been shown to serve as universal guardians for nucleic acids that activate TLRs, RIG-I-like receptors (RLRs), and DNA sensors of innate immune responses (Yanai et al., 2009; Kawai and Akira, 2010).

The transforming growth factor beta (TGF-β) signaling pathway has emerged as a possible target for modifying the polarization of macrophages during *Leishmania* infection, or the transition of M2 to M1 phenotype of macrophages. By balancing the classical activation of macrophages, blocking the TGF-beta signaling cascade can end up damaging the intracellular parasite (Zhu et al., 2011; Arase et al., 2014). Mothers against decapentaplegic homologs 1, 2, 3, and 4 (SMAD1, SMAD2, SMAD3, and SMAD4) are members of the SMAD family, where SMAD1-3 are receptor-regulated SMADs (R-SMADs). Several SMAD proteins make up the TGF-beta signaling pathway. The interaction of SMAD 4 (Co-SMAD) and SMAD2/SMAD3 (R-SMAD) is necessary for their translocation in the nucleus and binding to DNA elements for gene expression. The TGF-ligand phosphorylates several downstream SMAD proteins by binding to TGF-βRII. A putative target of miR-146a is SMAD4, and SMAD7 is a common inhibitory SMAD protein that suppresses R-SMAD activation and degrades TGF-RI. Blocking TGF, TGF-signaling-mediated gene expression might be possible by targeting SMAD 4 with miR-146a and increasing SMAD7 expression. Co-SMAD (SMAD4), a crucial signal transducer for TGF-receptors, is the target of the miR-146a. As a result, miR-146a will prevent the production of SMAD4, which will eventually prevent the TGF-signaling cascade from occurring because SMAD4 will not be present in the system (Nimsarkar et al., 2020).

More than 200 miRNAs, the majority of which belong to the let-7 family, are differentially expressed in CD4^+^ T cells after *L. donovani* infection. Most of the miRNAs that are increased target transcription factors that are predominantly controlling the IFN-γ pathway and are involved in polarizing TCD4 differentiation (Kumar et al., 2020). The fact that IFN-γ plays a crucial part in preventing *Leishmania* infection increases the intriguing possibility that the parasite’s increased miRNA production may be a virulence tactic (Kima and Soong, 2013). Moreover, INF-γ also needs to be continuously expressed to control the *CXCL10* gene (also known as interferon-inducible protein-10), which is necessary for developing a strong protective response (Gupta et al., 2011). Hence, *L. donovani* might employ miRNAs to prevent IFN-γ activated macrophages from performing their normal tasks and maintain their intracellular survival.

Similarly, *L. major* also induces more than 300 miRNAs in infected macrophages, and approximately 20% of them are differentially expressed, such as hsa-let-7a, hsa-miR-23b, hsa-miR-25, hsa-miR-26a, hsa-miR-140, hsa-miR-132, hsa-miR-146a, hsa-miR-155, hsa-miR-199, and hsa-miR-210 in different host cells. Furthermore, hsa-miR-146a and hsa-miR-155 are effective for the modulation of both innate and adaptive immune responses (Lemaire et al., 2013; Geraci et al., 2015; Lee et al., 2016; Nimsarkar et al., 2020). It is significant to note that, in contrast to *L. donovani*, where transcription factors (TFs) and elements interact, the INF-γ route is the primary target of miRNA in *L. major*. This could explain why different *Leishmania* species respond to IFN-γ differently (Kima and Soong, 2013). As *L. major* may inhibit the IFN-γ response in macrophages by altering the genes involved in innate immunity, cell adhesion, and proteasomal degradation, it can thrive in conditions that are rich in IFN-γ (Dogra et al., 2007). Interestingly, *L. major* stimulates the expression of miRNAs that target genes connected to the MAPK signaling pathway, just like *L. donovani* does.

To overcome host defenses and promote parasite survival and reproduction, other *Leishmania* species, including *L. amazonesis*, *L. viannia*, and *L. infantum*, modify the expression of miRNAs (Diotallevi et al., 2018; Souza et al., 2021). Consequently, *Leishmania amazonensis* increases the expression of hsa-miR-294, hsa-miR-30e, and hsa-miR-302d while downregulating TNF-α, monocyte chemoattractant protein-1 (Mcp-1), and oxide synthase 2 (Nos2), which are essential proteins for suppressing *Leishmania* infection (Fernandes et al., 2019). Infected macrophages with *L. viannia* and *L. infantum* overexpress hsa-miR-346, which controls MHC and interferon-associated genes. Depending on the parasite and host species, *Leishmania* infection changes the expression of miRNAs in the host cell. Moreover, the *Leishmania*-modulated miRNAs encourage infection persistence by controlling genes related to host immune responses (Lemaire et al., 2013; Geraci et al., 2015; Tiwari et al., 2017; Diotallevi et al., 2018).

In almost all organisms that have the necessary collection of proteins for the RNA interference (RNAi) pathway, the RNAi techniques has considered an incredibly effective tool for the better understanding of gene function. As soon as RNAi was established in *T. brucei* (Ngo et al., 1998), research workers seized the opportunity and developed RNAi technology as the go-to technique for suppressing the expression of genes, either for specific genes or for the entire genome. However, RNAi potential for forward genetics screening in research on *T. brucei* and other protozoan parasites that are RNAi-positive has not yet been completely realized. Moreover, the genome-wide RNAi screening approach was pioneered in *T. brucei* (Morris, 2002). Recently, it was shown that several *Leishmania* species have functional RNAi. To date, the use of such screens for gene function discovery and drug target validation has been limited and more widely applied for mammalian cells, *Drosophila*, and nematodes only (Giunti et al., 2021).

## Conclusion

This study has made a concerted effort to investigate the antiviral potential of human miRNAs targeting centrin genes 1–5. The species *donovani* employing the RNA22, RNA22-hybrid, and miRDB targets prediction algorithms. According to our study’s findings, 34 human miRNAs specifically target centrin genes 1–5. The current research confirms that *L. monocytogenes*, centrin-3, and centrin-5 are present. Out of the 2,635 known human miRNAs, it was discovered that *L. donovani* was significantly targeted by eight and twelve. The centrin-3 and centrin-5 genes were exclusively targeted by human miRNA, hsa-miR-5193: nine cellular miRNAs—hsa-miR-205-5p, hsa-miR-1236-3p, hsa-miR-4516, hsa-miR-4273, hsa-miR-383-3p, hsa-miR-103a-2-5p, hsa-miR-588, hsa-miR-4701-5p, and hs. These miRNAs specifically target genes and networks involved in a wide range of biological processes, including fatty acid biosynthesis and degradation, EGFR tyrosine kinase inhibitor resistance, focal adhesion, TGF-beta signaling, adipocytokine signaling, p53 signaling, Hippo signaling, Th17 cell differentiation, ErbB signaling, and cell cycle. Most importantly, this research suggests that these nine human miRNAs (miR-205-5p, miR-1236-3p, miR-4516, miR-4273, miR-383-3p, miR-103a-2-5p, miR-588, miR-4701-5p, and miR-556-5p) may act as distinguishing leishmaniasis markers. Characterizing these parasite-specific miRNAs and their host targets is therefore critical for developing a greater understanding of the molecular pathophysiology of leishmaniasis disorders. Discovering and characterizing parasite-specific miRNAs having highly specialized activities associated with cellular processes in parasites may lead to realistic parasitic illness treatment possibilities. In future investigations, a more in-depth exploration of the functional implications of the identified miRNAs on *Leishmania donovani* centrin genes could significantly contribute to our comprehension of the host–parasite interaction. Furthermore, delving into the therapeutic potential of manipulating these miRNAs may open avenues for innovative strategies in crafting targeted interventions for leishmaniasis.

## Data Availability

The original contributions presented in the study are included in the article/Supplementary Material; further inquiries can be directed to the corresponding author.
